# Dietary Diversity and Associated Factors among Children Aged 6-59 Months in Ethiopia: Analysis of Ethiopian Demographic and Health Survey 2016 (EDHS 2016)

**DOI:** 10.1155/2020/3040845

**Published:** 2020-08-21

**Authors:** Ataklti Gebretsadik Woldegebriel, Abraham Aregay Desta, Gebremedhin Gebreegziabiher, Asfawosen Aregay Berhe, Kiros Fenta Ajemu, Tewolde Wubayehu Woldearegay

**Affiliations:** ^1^Tigray Health Research Institute, Mekelle, Tigray, Ethiopia; ^2^Adigrat University, Adigrat, Tigray, Ethiopia

## Abstract

**Background:**

Dietary diversity is one of the key elements of diet quality. Even though different measures were taken to increase dietary diversity feeding practice in Ethiopia, the problem still remains high. Therefore, this study was done to identify determinants of inadequate minimum dietary practice among children aged 6-59 months in Ethiopia.

**Method:**

Secondary analysis of the data from the 2016 Ethiopian Demographic and Health Survey was done on a weighted sample of 5161 children aged 6-59 months. Data analysis was done using STATA v.14. Variables with *P* value < 0.05 in the bivariable analysis were candidates for the multivariable analysis to identify independent determinants of dietary diversity. Odds ratios (OR) were calculated at 95% confidence interval (CI).

**Results:**

A total of 5161 children aged 6 to 59 months were enrolled in the study. Only 8.5% of the children had the recommended minimum dietary diversity. Mother's education (adjusted odds ratio (AOR) = 2.51 (1.65, 3.83)), mothers currently working (adjusted odds ratio (AOR) = 1.83 (1.47, 2.29)), mother's wealth index (adjusted odds ratio (AOR) = 4.75 (3.31, 6.81)), age of a child (adjusted odds ratio (AOR) = 1.72 (1.24, 2.39)), and number of under-five children (adjusted odds ratio (AOR) = 1.49 (1.12, 2.00)) were significantly associated with the minimum dietary diversity.

**Conclusion:**

The minimum dietary diversity was not achieved by most children 6-59 months of age in Ethiopia. Ensuring large-scale interventions that focus on the identified factors should be considered by concerned bodies to improve the dietary diversity practice.

## 1. Background

Globally, malnutrition remains a serious burden of a public health concern. It affects social and economic development. It accounts directly or indirectly for 45% of the 5.9 million deaths of under-five children in 2015 [1]. This is the reason why adequate nutrition is identified as one of the pillars of public health interventions worldwide [2].

South Asia and sub-Saharan Africa are the home of a majority of the world's chronically undernourished children than elsewhere in the world [3]. Consequently, over 200 million African children under the age of five years suffer from malnutrition and fail to reach their full cognitive potential [4]. In Ethiopia, a child undernutrition is a major public health problem. The prevalence of stunting in under-five children in Ethiopia is 37% [5].

The main cause of malnutrition at the individual level is inadequate dietary diversity that does not provide adequate calories and micronutrients [6]. Micronutrient deficiency is a risk to children and is referred to as hidden hunger [7]. It is caused by chronic deficiency of vitamins and minerals as a consequence of nutrient inadequacy [8, 9].

Dietary assessment is an essential component of nutritional adequacy and provides information about the quantity and quality of food consumed in terms of nutrient adequacy and also consumption patterns and behaviors of the people [10]. It is a quantification of the number of different foods or food groups consumed over a reference time period [11]. The dietary diversity score has been identified as a potentially useful indicator of nutrient adequacy of children's diets [12–14]. This score can be used to assess the macro- and micronutrient consumption levels [15, 16].

The World Health Organization (WHO) recommends that children should consume foods from among the seven food groups [17]. Infant and Young Child Feeding (IYCF) practices recommend that children aged 6–23 months on breastfeeding should consume 4 or more other food groups daily [18]. Nonbreastfed children should consume milk or milk products, in addition to the four or more food groups [19].

The previous studies state about determinants of inadequate diet diversity practice, studies from Bangladesh [20], Dejen District, North West Ethiopia [21], and Malawi: findings from nationally representative data [22] showed that younger children and children from the poor household were less likely to meet the minimum dietary diversity. From the study finding in Myanmar [23] and Ghana [24], children from the illiterate mothers were less likely to meet the minimum dietary diversity. Despite poor dietary diversity which was visible among 6-59-month-old children in Ethiopia, however, a previous study in Ethiopia had identified determinants of dietary diversity among 6-23-month-old children [25].

Likewise, pouch studies were conducted on determinants of dietary diversity among 6-59-month-old children in various settings of the country and most shreds of evidence lack consistency and unrepresentative samples. Hence, until now, there was no population-based study permitting generalization about the determinant of dietary diversity among children 6-59 months. Therefore, this community-based and the national wide study was conducted to assess the determinant factors of minimum dietary diversity among age of six up to fifty-nine-month children. The result of this study will be used for national-level policymaking and programming by concerned bodies to intervene the identified gaps.

## 2. Methods

### 2.1. Study Setting

Ethiopia is located in the Horn of Africa. It is bordered by Eritrea to the north, Djibouti and Somalia to the east, Sudan and South Sudan to the west, and Kenya to the south. It has a high central plateau that varies from 1290 to 3000 m (4232 to 9843 ft) above sea level, with the highest mountain reaching 4533 m (14,872 ft). Ethiopia is home to about 13 million children under 5 years of age—approximately 16 percent of the total population of 96 million.

### 2.2. Data Source

The data source is nationally representative 2016 Ethiopian Demographic and Health Survey (EDHS) of children aged 6-59 months and their mothers. The survey was designed to provide population and health indicator estimates at the national, regional, and residence levels. The EDHS used a two-stage cluster sampling design with rural-urban and regions as strata yielding 21 sampling strata.

In the first stage, a total of 645 enumeration areas (EAs) were selected. For this study, child and women's data were extracted from the EDHS 2016 dataset. This analysis was restricted to the 5146 children aged 6-59 months having no missing value. The dataset used in this analysis contained information on the food items which was used to calculate the dietary diversity score.

### 2.3. Assessment of Dietary Diversity

The dietary quality of children was assessed using dietary diversity score. To measure dietary quality, we adopted the FAO 2011 Infant and Young Children Feeding (IYCF) guidelines. This was designed to measure dietary diversity for both breastfed and nonbreastfed children. The food items were categorized into seven major food groups based on the guidelines [26]. Structured 24-hour dietary recall of mothers was used to assess the foods consumed by the children.

### 2.4. Variables

#### 2.4.1. Outcome Variable

The minimal dietary diversity is defined as the proportion of children aged 6–23 months who consumed at least four food groups out of the seven referenced food groups within a 24-hour time. These food groups are (1) grains, roots, and tubers; (2) legumes and nuts; (3) dairy products; (4) flesh foods (meats/fish/poultry); (5) eggs; (6) vitamin A-rich fruits and vegetables; and (7) other fruits and vegetables [27].

The group scores were then summed to obtain the dietary diversity score, which ranges from zero to seven, where zero represents nonconsumption of any of the food items and seven represents the highest level of diet diversification. Children who took at least four food groups in the last 24 hours before the interview were considered to have achieved the adequate minimum dietary diversity (MDD). Therefore, the outcome variable was categorized as 0 = adequate MDD and 1 = inadequate MDD.

#### 2.4.2. The Independent Variables

The independent variables were identified from various literatures and grouped as characteristics of the family/household, child, parental, healthcare services, and the community. The child characteristics included sex, age, birth order, and having common childhood illnesses. The paternal characteristics included each parent's educational level, literacy level, working status, and maternal age marital status.

The wealth index and exposure to media were considered key household characteristics, whereas the place of residence (urban or rural) was considered a community-level variable. As health service characteristics, antenatal visits, place of delivery, and time of post natal care were included in this study.

## 3. Data Management and Analysis

Data analysis was carried out using STATA v.14; descriptive statistics were used to provide sample characteristics, including sociodemographic characteristics, individual, parental, household, healthcare, and community-level characteristics. Secondly, bivariate analysis between each explanatory variable and the outcome variable was done to determine the variables to include in the multivariable model.

Explanatory variables that were significantly associated with the outcome variable at *P* value less than 5% were included in the multivariable logistic regression model to identify independent determinants of MDD. Odds ratios (OR) were calculated to determine the strength of associations between independent variables and the outcome variable at 95% confidence interval (CI).

Sample weights were applied to compensate for the unequal probability of selection between the strata, which has also been geographically defined for nonresponses. A detailed explanation of the weighting procedure can be found in the EDHS methodology report [28]. We used “svy” in STATA v.14 to weight the survey data and perform the analyses. Variance inflation factor (VIF) > 10 was considered having multicollinearity effect.

## 4. Results

### 4.1. Sociodemographic Characteristics of Mothers

A total of 5161 children aged 6 to 59 months were enrolled along with their mothers in the study. The mean age (±SD) of the mothers was 27 ± 9.16 years. Out of the total mothers interviewed, almost half (2761) (53.5%) belong to the age group of 25–34 years. Two-thirds (3351) (64.9%) of the mothers' educational status were illiterate. Three-fourth (3889) (75.4%) of the mothers were not working at the time of data collection. (Table 1).

### 4.2. Household- and Community-Related Factors

Greater than two-thirds of the household (3853) (74.5%) have greater than four number of household members, no radio (3855) (74.7%), and male household head (4103) (79.5%). More than three-fourths of the mothers (1981) (38.4%) were poorest and less than one-fourth 956 (18.5%) were richest wealth index. More than one-fourth of mothers (892) (31.6%) were not able to visit antenatal care in the health facilities. Majority of fathers are currently working (4635) (89.8%). Majority of the community were rural residence (4243) (82.2%), and greater than half of the community 2563 (57.2%) were using unsafe drinking water (Table 1).

### 4.3. Child Characteristics

Nearly equal number of female (2590) (50.2%) and male (2571) (49.8%) children participated in the study. The mean (±SD) age was 26.6 months (SD ±2.1).Breastfeeding is common in Ethiopia, and most children (4451) (86.2%) were still breastfed during their second year of life. From the total of children, 1905 (37%) of them were in the age group 12-23 months. More than two-thirds (3710) (71%) of the households have less than three number of children. More than half (2790) (54.1) of children in this study were not taking vitamin A in the last 6 months (Table 2).

### 4.4. Frequency of Dietary Diversity

**Figure 1:**
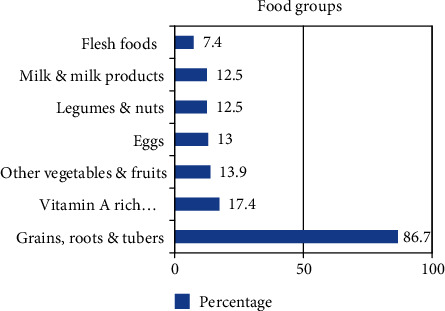
Food groups consumed among 6-59-month-old children in the last 24 hours, Ethiopian Demographic and Health Survey 2016 (*n* = 5161).

The dietary diversity determined based on a 24 h recall method showed that only 439 (8.5%) of the children had received the recommended minimum dietary diversity (children who fed four or more food items within 24 h preceding data collection) (Table 2). Grains, tubers, and roots (4490) (86.9%) were consumed by the greatest number of children within 24 hours preceding the survey. However, the intake of flesh food or organ meat was low, of the total children, only 392 (7.4%) (Figure 1).

**Figure 2:**
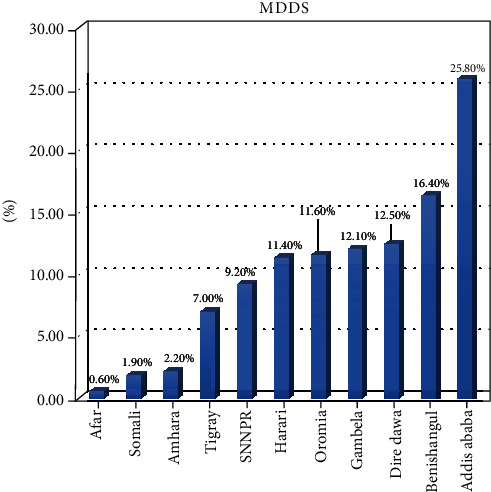
Regional variation of MDDS, Ethiopian Demographic and Health Survey 2016 (*n* = 5161).

**Figure 3:**
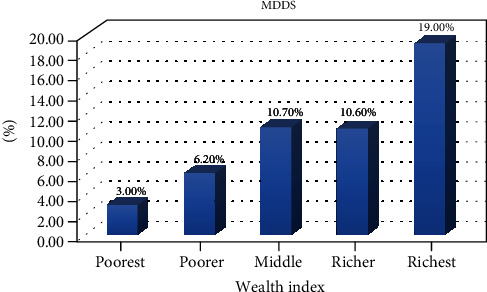
Wealth index of the households and MDDS, Ethiopian Demographic and Health Survey 2016 (*n* = 5161).

The dietary diversity determined based on a 24 h recall method among children showed that there were great variations among different regions of Ethiopia. Children in Addis Ababa (capital city) had received the highest recommended minimum dietary diversity (25%), and children in the following regions had received the least recommended minimal dietary diversity: Afar (0.6%), Somali (1.9%), Amhara (2.2%), and Tigray (7%) (Figure 2). Children born from mothers who had the highest wealth index have high percentage of using minimal dietary diversify food groups than those who had the poorest wealth index (Figure 3).

### 4.5. Factors Which Predict Dietary Diversity

In the binary logistic regression, mother's educational status, husband's educational status, mother currently working, residence, wealth index, child age, birth order, the birth interval of the child, number of under-five children, the household has radio, and the household has television were significantly associated.

The study found that children born from mothers who had the highest education level (adjusted odds ratio (AOR) = 2.51 (1.65, 3.83)) had greater odds of feeding diversified foods. Another most vital factor significantly associated with minimum dietary diversity was the age of a child. Children aged 12–23 months ((AOR 1.72; 95% CI (1.24, 2.39)) have a minimum dietary diversity compared to children aged 6–11 months.

It was found that mother's working status had a significant association with dietary diversity. Children born from mother currently working (AOR 1.83; 95% CI (1.47, 2.29)) have a higher odds of having the minimum dietary diversity compared with those born from mother not currently working.

This study also indicated that wealth index had a significant association with dietary diversity. Children born from mother's wealth index of richest (AOR 4.75; 95% CI (3.31, 6.81)), richer (AOR 3.23; 95% CI (2.19, 4.77)), middle (AOR 3.63; 95% CI (2.52, 5.23)), and poorer (AOR 1.95; 95% CI (1.32, 2.89)) have higher odds of having the minimum dietary diversity compared with those born from poorest mothers.

Furthermore, this study revealed that the number of under-five children in the household had a significant association with dietary diversity. The number of children less than three in the household (AOR 1.49; 95% CI (1.12, 2.01)) has higher odds of having the minimum dietary diversity compared with greater than three number of children (Table 3).

## 5. Discussion

This community-based cross-sectional study assessed determinants of minimum dietary diversity among children aged 6-59 months in Ethiopia. The main goal of dietary diversity is to promote households to consume diversified diets rather than consuming repetitive diets throughout twenty-four hours.

This study revealed that only 8.5% of children received minimum dietary diversity throughout twenty-four hours. The finding is similar to a study done in Dembecha, northwest Ethiopia (8.6%) [29], and Gorche District, Southern Ethiopia (10.6%) [30].

Furthermore, it is higher than the study in Kenya (6.8%) [31]. However, it is lower compared to the study conducted in Rwanda and Burundi Demographic and Health Surveys (Rwanda 23%, Burundi 16%) [32]; Tanzania [33]; Addis Ababa, Ethiopia 59.9% [34]; and Morondava (47.6%) and Moramanga (42.1%) districts of Madagascar, respectively [35].

The differences might be due to the fact that this study was community-based; the overall estimate of minimum dietary diversity could be lower than small-scale and health facility-based studies. These differences might be also due to variations in socioeconomic status, dietary habit, culture, study design, and self-reported measurement, and recall method could also affect the estimated minimum dietary diversity.

Children born from illiterate mothers were less likely to practice minimum dietary diversity.

It was concurrent with a study conducted in Wolaita Sodo town, Southern Ethiopia [36], Northwest Ethiopia [37], and urban Zambia [38]. We also show that a low education level of mothers increased the likelihood of having a low dietary diversity score. Similar report is also observed in Madagascar and Afar, North Ethiopia [35, 39].

The possible reason might be as the education level of the mother increased and as the mother engaged in paid work, there is access to more information on educational messages and different mass media like radio, television, and newspaper. They also participate actively in health education sessions and child feeding demonstrations in health facilities; as a result, their children are more likely to fulfill the minimum dietary diversity requirement.

Another important determinant of the minimum dietary diversity of children in the study area is household economical status. Similar results are observed in Nepal DHS (26%) [40], Dejen District, North West Ethiopia, and the Philippines [21, 41]. This may indicate that family income has a direct association with household food security, since food consumption is believed to be heavily influenced by income of the household.

There is also a regional variation for the requirement of minimum dietary diversity score among 6-59-month-old children in Ethiopia. This might be due to differences in agroecology, feeding habits, lifestyles, and demography among the regions. The places with the highest minimum dietary diversity score are in Addis Ababa, Ethiopia, and lowest in nomadic regions (Afar and Somali). This might be because Addis Ababa is located in the urban center, the capital city of Ethiopia; mothers' might have easy access to information, market, and health services about dietary diversity and child feeding practices.

Children age 12–23 months were significantly associated with minimum dietary diversity. This might be due to infant's mothers during 6–11 months who did not introduce solid and semisolid food; they are only introducing simply feeding milk along with breast milk. Many children in the household have less minimum dietary diversity intake than older children.

In this study, the number of children in the household is significantly associated with minimum dietary diversity. A possible explanation might be families with many numbers of children are less likely to purchase diversified food groups and unable to fulfill their children's dietary requirement.

### 5.1. Limitation of the Study

The major limitation of this study was its cross-sectional design, which does not allow the identification of the precedence in time between exposure and end point.

## 6. Conclusion

Less than one-fifth of the children aged 6–59 months received minimum dietary diversity in Ethiopia. Mother's education level, mothers' wealth index, mothers' working status, age of a child, and number of under-five children were the factors statistically associated with minimum dietary diversity of children aged 6–59 months. Interventions which focus on improving the socioeconomic status of households, educational status of mothers, and diversified food consumption of children aged 6-59 months should be strengthened.

## Figures and Tables

**Table 1:** Sociodemographic characteristics and other characteristics of the mothers, EDHS 2016.

Variables	Un weighted frequency	Un weighted percentage	Weighted frequency
Maternal			
Age of mothers			
<20	197	3.8	193
20-29	2761	53.5	2804
30-39	1906	36.9	2010
≥40	297	5.8	334
Mother's educational level			
Illiterate	3351	64.9	3523
Primarily	1295	25.1	1481
Secondary	335	6.5	229
Higher	180	3.5	108
Marital status			
Never in union	24	0.5	21
Living with partner	4920	95.3	5089
Widowed	41	0.8	57
Divorced	42	0.8	36
No longer living together/separated	98	2.6	91
Religion			
Orthodox	1362	26.4	1454
Catholic	32	0.6	33
Protestant	835	16.1	821
Muslin	2837	55.0	2955
Other and traditional	95	1.9	35
Mothers currently working			
No	3889	75.4	4030
Yes	1272	24.6	1311
ANC visit			
None	892	31.6	1017
1-3	843	29.9	901
>4	1085	38.5	1030
Community related			
Residence			
Urban	918	17.8	523
Rural	4243	82.2	4818
Wealth index combined			
Poorest	1981	38.4	1396
Poorer	889	17.2	1226
Middle	731	14.2	1152
Richer	604	11.7	894
Richest	956	18.5	673
Source of drinking water			
Nonimproved	2563	50.3	2216
Improved	2598	49.7	3125

**Table 2:** Sociodemographic characteristics and other characteristics of the mothers and children, EDHS 2016.

Variables	Categories	MDDS	Odds ratio (95% CI)
Age of the child in months			
6-11	1014	19.6	1065
12-23	1907	37.0	1988
24-35	460	8.9	438
36-47	886	17.2	906
Sex of the child			
Male	2571	49.8	2614
Female	2590	50.2	2727
Birth interval of the child			
First order	1084	21.0	1030
<24months	1049	20.3	974
≥24months	3028	58.7	3337
Birth order			
≤3	2779	53.8	2713
>3	2382	46.2	2628
Number of under-5 children			
<3	3710	71.9	4005
≥3	1451	28.1	1336
Minimal dietary score			
≤3	4722	91.5	4853
≥4	439	8.5	488
Currently breast feeding			
Yes	4451	86.2	4748
No	710	13.8	593
Had diarrhea recently			
No	4510	87.4	4622
Yes, last two weeks	643	12.5	711

**Table 3:** Bivariate and multivariable logistic regression between different level predictors and minimum dietary diversity of children aged 6–59 months, EDHS 2016.

Variables	Categories	MDDS		Odds ratio (95% CI)			
		<4 (*n* (%)	≥4 (*n* (%)	COR (95% CI)	*P* value	AOR (95% CI)	*P* value
Mother's educational status	Illiterate	196 (5.8)	3155 (94.2)	1		1	
	Primary	134 (10.3)	1161 (89.7)	1.858 (1.476, 2.339)	<0.001^∗^	1.270 (0.989, 1.631)	0.061
	Secondary	51 (15.2)	284 (84.8)	2.891 (2.076, 4.025)	<0.001^∗^	1.322 (0.900, 1.941)	0.155
	Higher	58 (32.2)	122 (67.8)	7.653 (5.424, 10.798)	<0.001^∗^	2.514 (1.651,3.829)	<0.001^∗^

Husband's educational status	No education	121 (5.0)	2322 (95.0)	0.222 (0.162, 0.305)	<0.001^∗^	0.759 (0.486, 1.184)	0.224
	Primarily	151 (9.2)	1492 (90.8)	0.432 (0.317, 0.588)	<0.001^∗^	0.924 (0.613, 1.393)	0.706
	Secondary	74 (14.8)	427 (85.2)	0.740 (0.517, 1.057)	0.098	1.141 (0.758, 1.717)	0.528
	Higher	71 (19.0)	303 (81.0)	1			

Age of mothers at birth	<20	16 (8.1)	181 (91.9)	1			
	20-29	228 (8.3)	2533 (91.7)	1.018 (600, 1.728)	0.947		
	30-39	173 (9.1)	1733 (90.9)	1.129 (0.662, 1.927)	0.656		
	≥40	22 (7.4)	275 (92.6)	0.905 (0.463, 1.770)	0.771		

Mother currently working	Not working	252 (6.5)	3637 (93.5)	1		1	
	Working	187 (14.7)	1085 (85.3)	2.487 (2.035, 3.041)	<0.001^∗^	1.833 (1.468, 2.288)	<0.001^∗^

Residence	Rural	269 (6.3)	3974 (93.7)	1		1	
	Urban	170 (18.5)	748 (81.5)	3.358 (2.729, 4.131)	<0.001^∗^	1.307 (0.865, 1.974)	0.204

Wealth index	Poorest	60 (3.0)	1921 (97.0)	1		1	
	Poorer	55 (6.2)	834 (93.8)	2.111 (1.451, 3.071)	<0.001^∗^	1.955 (1.322, 2.890)	0.001^∗^
	Middle	78 (10.7)	653 (89.3)	3.824 (2.700, 5.417)	<0.001^∗^	3.631 (2.519, 5.233)	<0.001^∗^
	Richer	64 (10.6)	540 (89.4)	3.795 (2.634, 5.466)	<0.001^∗^	3.235 (2.195, 4.766)	<0.001^∗^
	Richest	182 (19.0)	774 (81.0)	7.528 (5.558, 10.198)	<0.001^∗^	4.746 (3.308, 6.809)	<0.001^∗^

Child age (months)	6-11	80 (7.9)	934 (92.1)	1.307 ((0.915, 1.865)	0.141	1.078 (0.738, 1.575)	0.697
	12-23	227 (11.9)	1680 (88.1)	2.061 (1.518, 2.799)	<0.001^∗^	1.720 (1.240, 2.387)	0.001^∗^
	24-35	23 (5.0)	437 (95.0)	0.803 (0.487, 1.324)	0.390	0.991 (0.582, 1.688)	0.974
	36-47	54 (6.1)	832 (93.9)	0.990 (0.672, 1.459)		1.079 (0.715, 1.628)	0.717
	48-59	55 (6.2)	839 (93.8)	1		1	

Sex of the child	Male	211 (8.2)	2360 (91.8)	0.926 (0.762, 1.126)	0.443		
	Female	228 (8.8)	2362 (91.2)	1			

Birth order	≤3	281 (10.1)	2498 (89.9)	1			
	>3	158 (6.6)	2224 (93.4)	0.632 (0.515, 0.774)	<0.001^∗^	1.008 (0.775, 1.311)	0.951

Birth interval of the child	First order	126 (11.6)	958 (88.4)	1			
	<24 months	59 (5.6)	990 (94.4)	0.453 (0.328, 0.625)	<0.001^∗^	0.889 (0.607, 1.302)	0.889
	≥24months	254 (8.4)	2774 (91.6)	0.696 (0.555, 0.873)	0.002^∗^	0.932 (0.716, 1.214)	0.932

Number of under 5 children	<3	367 (9.9)	3343 (90.1)	12.103 (1.621, 2.728)	<0.001^∗^	1.497 (1.118, 2.004)	0.007^∗^
	≥3	72 (5.0)	1379 (95.0)	1		1	

HH has radio	Yes	163 (13.0)	1094 (87.0)	1		1	
	No	276 (7.1)	3628 (92.9)	0.511 (0.416, 0.627)	<0.001^∗^	0.812 (0.645, 1.023)	0.812

HH has television	Yes	151 (21.5)	552 (78.5)	1			
	No	288 (6.5)	4170 (93.5)	0.252 (0.203, 0.313)	<0.001^∗^	0.719 (0.486, 1.063)	0.719

Sex of HH head	Male	359 (8.7)	3744 (91.3)	1			
	Female	80 (7.6)	978 (92.4)	0.853 (0.663, 1.098)	0.217		

Maximum SE 0.272; Hosmer and Lemeshow 0.169; COR: crude odds ratio; AOR: adjusted odds ratio.

## Data Availability

The data used to analyze the current study are available from the corresponding author upon reasonable request.

## Abbreviations

AOR: Adjusted odds ratio
EA: Enumeration areas
EDHS: Ethiopian Demographic and Health Survey
MDDS: Minimum dietary diversity score
IYCF: Infant and Young Child Feeding
VIF: Variance inflation factor.
